# Hepatic metastasis from perianal Paget’s disease without identified underlying carcinoma: a case report

**DOI:** 10.1186/s12957-021-02439-4

**Published:** 2021-12-02

**Authors:** Yi-Sheng Cao, Shu-Yan Wang

**Affiliations:** 1 Department of Colorectal Surgery, HwaMei Hospital, University of Chinese Academy of Sciences, No. 41 Northwest Street, Ningbo, Zhejiang, 315000 China; 2 Ningbo Institute of Life and Health Industry, University of Chinese Academy of Sciences, No. 41 Northwest Street, Ningbo, Zhejiang, 315000 China; 3 Key Laboratory of Diagnosis and Treatment of Digestive System Tumors of Zhejiang Province, Ningbo, 315000 China; 4 Ningbo Pathological Diagnosis Center, Ningbo, 315000 China

**Keywords:** Perianal Paget’s disease, Hepatic metastasis, Underlying carcinoma, Case report

## Abstract

**Background:**

Perianal Paget’s disease (PPD) is a rare malignancy, often associated with an underlying adenocarcinoma and a poor prognosis.

**Case presentation:**

A 69-year-old female was presented with a history of perianal pruritus for 6 months and enlarged inguinal lymph nodes in the left side. Paget cells were confirmed by pathology after a wide excision of perianal skin. Radiotherapy was performed covering the bilateral inguinal lymphatic drainage area. Hepatic metastasis was found 8 months after surgery. Hepatic artery embolization (HAE) and high-intensity focused ultrasound therapy (HIFU) were performed successively. However, hepatic metastasis happened again 3 months later. Ultrasound-guided percutaneous radiofrequency ablation (PRFA) was carried out and various means of inspection could not identify the primary tumor. In the case of rapid progression of the tumor, we gave the patient chemotherapy regimens of XELOX. After 4 cycles of chemotherapy, the tumor marker went down continuously and the hepatic metastasis stayed stable.

**Conclusions:**

Hepatic metastasis from perianal Paget’s disease without identified underlying carcinoma may benefit from XELOX on the basis of adenocarcinoma.

## Background

Paget’s disease was an uncommon intraepithelial adenocarcinoma named by Sir James Paget describing breast cancer patients with characteristic lesions around the nipples in 1874 [1]. While perianal Paget’s disease (PPD) was firstly reported in 1893, which accounted for 20% of extramammary Paget’s disease cases that usually happened in vulva, perineum, penis, scrotum, and axilla [2]. Those patients may have a high incidence of underlying primary carcinoma, which often refers to a poor prognosis. We report a rare case of hepatic metastasis from PPD without underlying carcinoma detected and discuss the possibly effective therapy.

## Case presentation

**Fig. 1:**
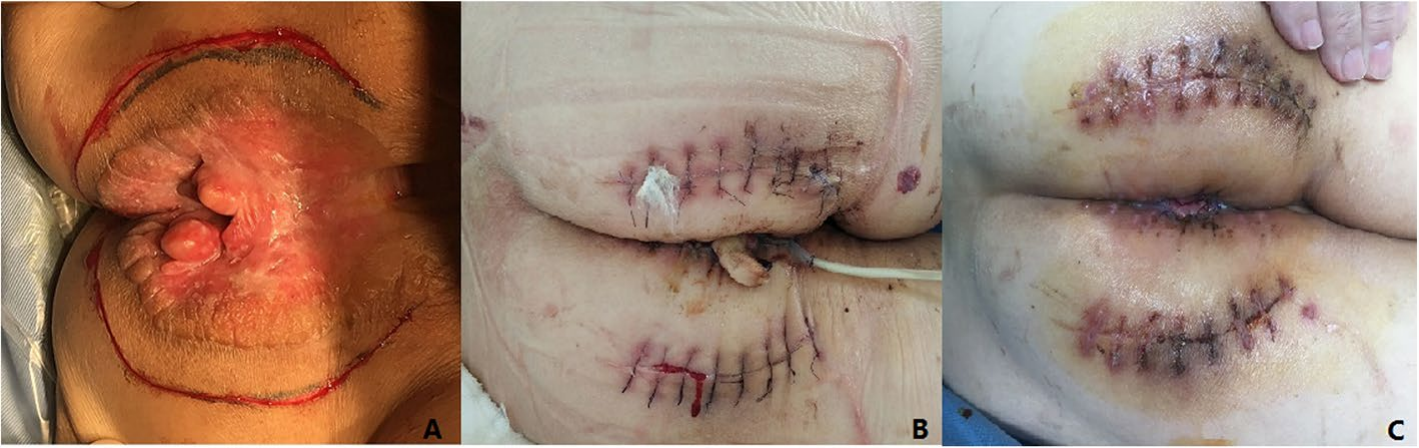
A raised and raw hyperaemic area 8 cm in diameter surround the anal (**A**). Perianal flap transfer was performed to cover the perianal skin defect (**B**) and the wound healed well 1 month after surgery (**C**)

**Fig. 2:**
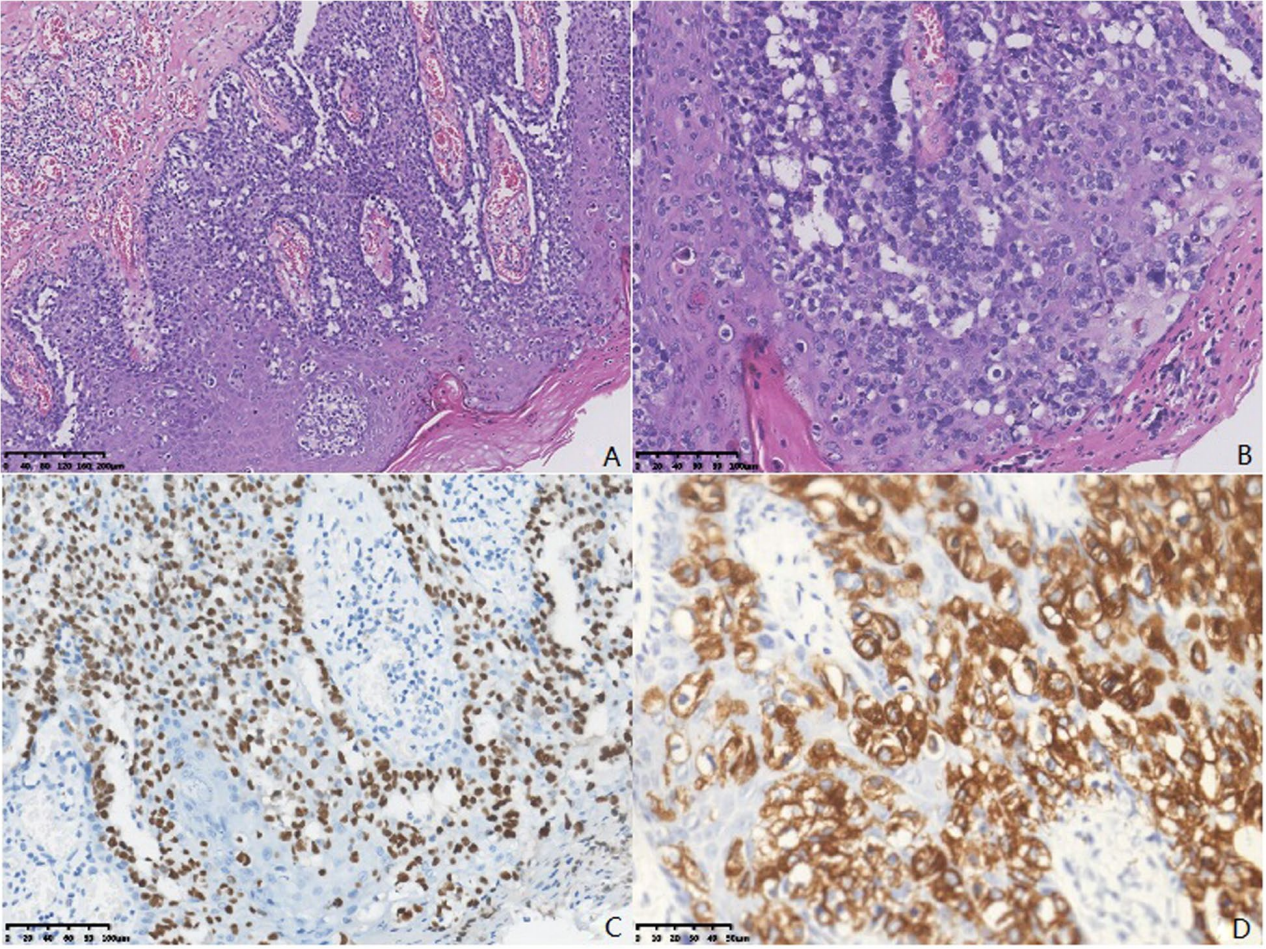
The Paget’s cell is characterized by large volume, rich and transparent cytoplasm, and the round, large hyperchromatic nuclei, with mitotic figures (**A**). Immunohistochemical analysis was positive in CDX-2 (**B**) and CK20 (**C**), which strongly suggested the possible origin of adenocarcinoma. Heteromorphic solid cell nests were observed in the hepatic penetrating tissues. Liver biopsy showed large cell body, little cytoplasm, hyperchromatic nuclei with distinct atypia, and the mitotic figures were visible, as well as intracellular mucous (**D**)

**Fig. 3:**
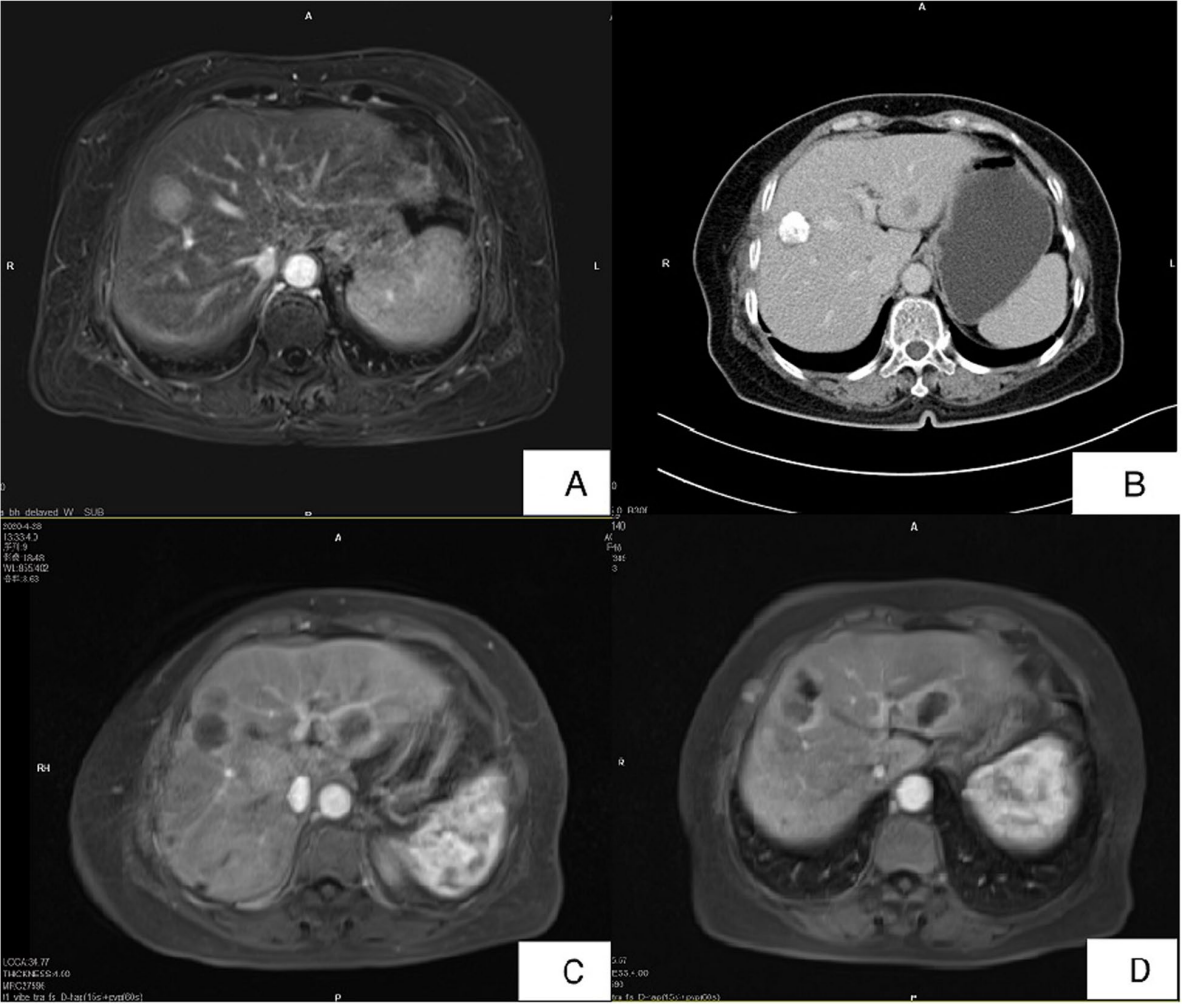
The size change of hepatic metastasis judging by images from 2019-12-29 (**A**), 2020-03-05 (**B**), 2020-04-28 (**C**), and 2020-07-29 (**D**)

**Fig. 4:**
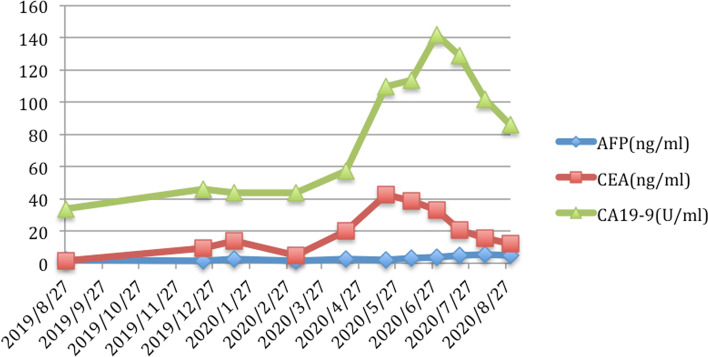
Trend of the tumor marker

A 69-year-old female was presented to the hospital in April 2019 with a history of perianal pruritus for 6 months. Physical examination showed a raised and raw hyperemic area 8 cm in diameter surround the anal and enlarged inguinal lymph nodes in the left side. The patient had neither weight loss nor gastrointestinal symptoms and had no other history of surgery except local resection for benign breast lobular hyperplasia 30 years ago. The patient’s tumor markers, such as carcinoembryonic antigen (CEA) and carbohydrate antigen 199 (CA199), were normal. Abdominal enhanced computerized tomography (CT) showed multiple intrahepatic cysts and perianal skin thickening, with lymph nodes as large as 35 × 19 mm in the left inguinal region. Chest high-resolution CT (HRCT) reveals a 5-mm ground glass nodule with a clear boundary in the posterior segment of the upper lobe tip of the left lung (LUNG-RADs 3). Both of the gastroscopy and colonoscopy had no positive findings. Ultrasound exam and mammography of the breast were also normal. Local biopsy conformed with the diagnosis of Paget’s disease. A wide excision of perianal skin (Fig. 1A) and temporary loop stoma of the transverse colon was performed. Post-operation pathology confirmed the diagnosis of Paget’s disease with negative surgical margin (Fig. 2A, B), and the tumor was as large as 7.5 cm × 5.0 cm × 0.2 cm, without any infiltration or vessel invasion. Immunohistochemical analysis was positive in caudal type homeobox 2 (CDX-2) (Fig. 2 C) and cytokeratin 20 (CK20) (Fig. 2D), and negative in GCDFP-15, S-100, and Her-2. Due to large skin defect after excision of the lesion, perianal flap transfer was performed. After her wound healed (Fig. 1B, C), the patient began to receive radiotherapy covering the bilateral inguinal lymphatic drainage area. In December 2019, the abdominal enhanced CT showed a nodule in slightly low density in IV–VII segment of the liver, 21 × 21 mm in size during follow-up (Fig. 3A). Magnetic resonance imaging (MRI) revealed the nodule in VII segment of the liver to be the long T1 and T2 signal one, as well as nodules in segment III and VII, 4–5 mm in diam. Ultrasound-guided puncture confirmed the nodule to be poorly differentiated adenocarcinoma, positive in cell keratin 7 (CK7)、CK20 and CDX-2, and negative in Hepatocyte and CgA. Hepatic artery embolization (HAE) and high-intensity focused ultrasound therapy (HIFU) were performed successively. After those therapies, the patient’s tumor marker went down a bit but soon rose again. In March 2020, the abdominal CT scan showed the nodule in segment III was 12 × 15 mm in size, bigger than it was 3 months ago (Fig. 3B). One and a half months later, MRI found these nodules grew rapidly accompanied with some new ones, and the biggest one was as large as 41 × 33 mm (Fig. 3C). Ultrasound guided percutaneous radiofrequency ablation (PRFA) was carried out. Colonoscopy, chest CT scan and breast ultrasound were performed again to identify the primary tumor as well as PET-CT. Though there was still no positive finding, we gave the patient chemotherapy regimens of XELOX (Oxaliplatin 130 mg/m^2^, ivgtt D1, Xeloda 1000 mg/m^2^, po bid, D1–14, repeat every 3 weeks) since 22 May. After 4 cycles of chemotherapy, the tumor markers went down continuously (Fig. 4) and MRI found the patient in the status of partial response (PR) (Fig. 3D).

## Discussion

The PPD is rare and accounts for 5.4–20% of extramammary Paget’s disease (EMPD) cases [2–4], often associated with internal malignancies and a poor prognosis [5]. The number of recorded cases is small, most of which describe the disease manifestations, and a variety of treatment modalities. The most important diagnostic criteria for PPD is Paget’s cells, which are characterized by round ceils with a pale vacuolated cytoplasm and a large reticular nucleus [6]. A biopsy of the lesion should be implemented to confirm the diagnosis before surgery. The differential diagnosis should include leukoplakia, Bowen’s disease, squamous-cell cancer, eczema, and so on. In a review by Grow, PPD can be divided into three different patterns on the basis of origins, 50% of cases associated with an apocrine or eccrine carcinoma, called “original Paget’s disease (PD)”, 25% with an underlying anal/rectal adenocarcinoma or squamous cell carcinoma, called “pagetoid extension”, and 25% with no underlying malignant lesion [7]. Immunochemical analysis plays an important role in judging the origin of the PPD. Liao et al studied the clinicopathological and immunochemical features of 13 PPD cases, and found that GCDFP-15 was only expressed in primary PPD, while CDX-2 was only positive in secondary cases [8]. In our case, the immunochemical result of CDX-2, CK20, and GCDFP-15 indicated the patient suffered from secondary PPD around the anal without underlying carcinoma according to CT scans, gastroenteroscopy, PET-CT, and any other examination methods. The enlarged inguinal lymph nodes in the left side indicated a high degree of malignancy. The disease progressed months later, but no other primary tumor was found. According to the monistic principle, we believe that the metastasis originate from perianal Paget’s disease.

Treatment for PPD varies depending on the different origins and staging of the disease. It is commonly accepted that wide excision could direct toward achieving local control, especially for the “original PD”, and it can be performed repeatedly if recurrence happens. If the skin defect is huge after resection, local rotation flaps, or skin grafting might preserve patients’ anal function. For patients with downward spread of anal/rectal malignant, more aggressive surgery should be considered to cure the disease, including the possibility of an abdominoperineal resection (APR) [9]. For PPD patients with no underlying carcinoma, there is no widely accepted recommendation yet because of the small number of the cases. Wide excision is the first choice just like the one we performed, and a negative margin should be guaranteed. Patients must be closely followed up, to detect not only a possible recurrence, but also delayed underlying malignant tumor. Besides surgery, radiation therapy is an alternative treatment in some circumstances. Though there are no randomized controlled trials to compare surgery with radiation therapy for EMPD, radiation therapy may be indicated in patients medically unfit for surgery, for recurrence following surgery, in any patient who wishes to preserve the functional or as an adjuvant to surgery in patients with an underlying adenocarcinoma [10]. However, some literature contained a view that radiotherapy had no place in the management of the condition because of high recurrence rates [11, 12]. We gave the patient radiotherapy covering the bilateral inguinal lymphatic drainage area to prevent possible lymph node metastasis. Chemotherapy is another way of adjuvant treatment often combined with radiation, but there is no guideline for the deployment of chemotherapy drugs. Topical chemotherapeutic agents include 5-fluorouracil (5-FU) and mitomycin C, though no survival data has been recorded due to sporadic cases. In general, this is in the setting of invasive or more aggressive recurrent disease, because the response to chemotherapy has been poor. 5-FU may be useful for symptomatic relief, preoperative delineation of disease extent, cytoreduction prior to surgery and postoperative detection of early disease recurrence [13]. Some other agents, such as docetaxel combined with cisplatin, S-1 combined with docetaxel, combination of cisplatin-epirubicin-paclitaxel are reported to be used in the treatment of advanced cases [14–16]. Although some authors have reported successful treatment of Paget’s disease with chemo-radiotherapy, the use of adjuvant therapy has not been associated with improved local control or survival [17]. Based on our experience, the patient was treated with XELOX regimen according to pathological findings of adenocarcinoma from hepatic metastasis without any identified underlying carcinoma, and it worked, to a certain extent, from the descending tumor marker and the steady state of metastases.

Shutze et al. gave classification to PPD based on the disease pathology from the cases reported in the literature and correlated with surgical treatment [18]. The survival data varied dramatically from diseases in different stages. Wide local excision was recommended for patients with Paget’s cells found in perianal epidermis and adnexae without primary carcinoma (stage I) and cutaneous Paget’s disease with associated adnexal carcinoma (stage IIA), though the most common morbidity after surgery was local recurrence with the rates of 44–60% [5, 19]. But tumor stages are not fixed and sometimes require restaging as the disease progresses or the underlying carcinoma is identified. For patients with PPD of more aggressive staging, the prognosis is extremely poor. Distance metastases often occur months after the first treatment, usually involving the liver, the lung, and the bone, and may lead to rapid progress. Among three different patterns of PPD, the pagetoid extension has worse prognosis than original PD [20], while PPD without underlying malignant lesion has the most uncertain outcome. This patient had non-invasive PPD at first surgery except enlarged inguinal lymph nodes in the left side, but only 7 months since surgery, she was found to have hepatic metastasis, which recurred after HAE and HIFU. When distant metastasis happens, local treatment alone cannot obtain satisfactory effect. So, we gave her chemotherapy regimens of XELOX based on the pathologic findings of adenocarcinoma, and fortunately, the patient got a declining tumor marker value and stable metastases. Although the existence of other primary tumors has not been found up to now, liver metastases might come from other micro-adenocarcinoma, which can be further observed and discussed in follow-up.

## Conclusions

After radical resection of PPD, a thorough screening of the patient should be performed to detect the origin of the primary lesion and find any potential malignancy or metastasis, which often affects the clinical outcome. Although the role of chemotherapy in the management of PPD has not been fully assessed, combination chemotherapy such as XELOX may be a promising alternative for patients with advanced unresectable metastasis. Further prospective clinical trials with more cases are advised to validate the recommendations.

## Data Availability

The authors confirm the data and materials are available.

## Abbreviations

PPD: Perianal Paget’s disease
HAE: Hepatic artery embolization
HIFU: High-intensity focused ultrasound therapy
PRFA: Percutaneous radiofrequency ablation
CEA: Carcinoembryonic antigen
CA199: Carbohydrate antigen 199
CT: Computerized tomography
CDX-2: Caudal type homeobox 2
CK20: Cytokeratin 20
MRI: Magnetic resonance imaging
CK7: Cell keratin 7
EMPD: Extramammary Paget’s disease
PD: Paget’s disease
APR: Abdominoperineal resection
5-FU: 5-fluorouracil
